# Population Genetic Baseline of the First Plataspid Stink Bug Symbiosis (Hemiptera: Heteroptera: Plataspidae) Reported in North America

**DOI:** 10.3390/insects2030264

**Published:** 2011-06-24

**Authors:** Tracie M. Jenkins, Tyler D. Eaton

**Affiliations:** Department of Entomology, The University of Georgia, Griffin Campus, 1109 Experiment Street, Griffin, GA 30223, USA; E-Mail: eaton@uga.edu

**Keywords:** stinkbugs, *Megacopta cribraria*, *Candidatus Ishikawaella capsulate*, *Wolbachia*, symbiosis, mtDNA, nuDNA

## Abstract

The stink bug, *Megacopta cribraria*, has an obligate relationship with a bacterial endosymbiont which allows it to feed on legumes. The insect is a pest of soybeans in Asia and was first reported in the Western Hemisphere in October 2009 on kudzu vine, *Pueraria montana*, in North Georgia, USA. By October 2010 *M. cribraria* had been confirmed in 80 counties in Georgia actively feeding on kudzu vine and soybean plants. Since the symbiosis may support the bug's ecological expansions, a population genetic baseline for the symbiosis was developed from mitochondrial DNA (mtDNA) and nuclear DNA (nuDNA) gene sequence collected from each insect and its primary γ- proteobacterium and secondary α -proteobacterium endosymbionts. A single mitochondrial DNA haplotype was found in all insects sampled in Georgia and South Carolina identified as GA1. The GAI haplotype appears to be rapidly dispersing across Georgia and into contiguous states. Primary and secondary endosymbiont gene sequences from *M. cribraria* in Georgia were the same as those found in recently collected *Megacopta* samples from Japan. The implications of these data are discussed.

## 1. Introduction

Genetic impacts related to insect invasions such as horizontal gene transfer and interspecific hybridization have generally not been the emphasis of invasive biological study [1]. These phenomena are of some concern because of the impacts they could have on native insect genetic ecologies that directly affect urban and traditional agriculture. Insights into genetic impacts are in turn dependent on knowledge of the genetic baseline for an invasive species. The quicker the baseline is established, the faster genetic impacts can be determined. In October 2009 the first Asian plataspid stink bug reported in the Western Hemisphere, *Megacopta cribraria* (Fabricius 1798) (Hemiptera: Heteroptera), was discovered in the continental United States in north Georgia [2]. Eighteen months later, the first genetic characterization of the bug and its obligate endosymbiont on which its fitness depends [3] is herein reported and discussed.

**Figure 1:**
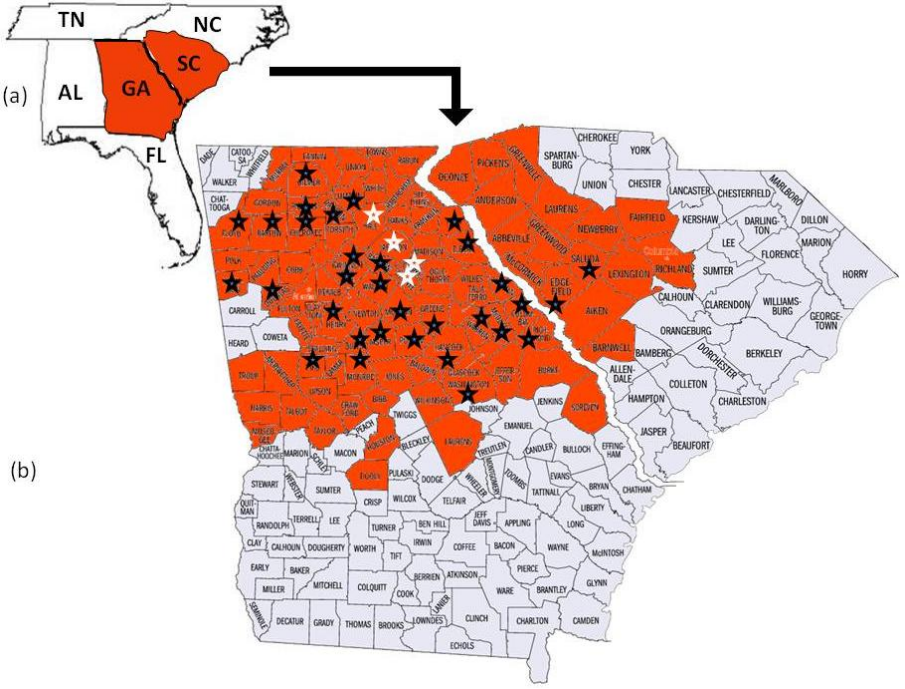
Map delineating area infected with the stinkbug, *Megacopta cribraria* as of November 2010. (**a**) Georgia (GA) and South Carolina (SC) with contiguous states identified: Tennessee (TN), North Carolina (NC), Florida (FL) and Alabama (AL). (**b**) The states GA and SC enlarged to show the counties in those states with the invasive stinkbug as of November 2010. All starred areas represent counties from which samples were analyzed. White stars represent the counties from which the stinkbug was initially identified and from which sample mitochondrial genomes were sequenced.

*Megacopta cribraria*, which is an agricultural pest species primarily of legumes in Asia [2], was reported when thousands of the smelly insects were found on the sides of two houses in Jackson County, Georgia, USA [2,4]. Identified from morphological characters [2] and DNA sequence [5], the established and geographically adaptive invasive [6] developed on kudzu, *Pueraria montana* var. lobata (Willd.) Ohwi (Fabaceae), before adults moved onto the sides of the houses in search of overwintering shelter [2,4]. Since their discovery, *Megacopta cribraria* have quickly reproduced to form large dispersing populations [4]. In November 2009, the insect was confirmed on kudzu vine in nine contiguous North Georgia counties north and east of lands used for soybean production [4]. By October 2010, prior to overwintering, *M. cribraria* had been confirmed in 80 counties in Georgia and 16 counties in South Carolina actively feeding on kudzu vine and soybean plants (Figure 1). Stinkbug sightings continued to be reported through November 2010 in Georgia, North Carolina, Tennessee, and Alabama. This is significant for two reasons. First in parts of Asia *Megacopta* are serious pests of soybeans [7,8]. They damage the young leaves, stems and newly developed soybean pods causing lesions to develop on the leaf surfaces and plant death [9]. Second, according to the USDA National Agricultural Statistics Service, 76,616,000 acres of soybeans valued at $38,915,328,000 were planted in the U.S. in 2010. Georgia appears to be the entry site of *M. cribraria*, which continues to spread across the state and into contiguous surrounding state ecologies where soybeans are grown. Genetic studies are ongoing in an effort to understand the adaptive potential of this bug. Insights into how it adapts may help to control its spread and ability to feed on soybeans.

The ability of the insect to feed on legumes has been largely attributed to a mutualistic symbiosis in which an obligate γ-proteobacterium, *Candidatus Ishikawaella capsulata* [3,10], serves to increase host levels of essential amino acids, which in turn increases insect fertility and fecundity [11,12]. The increased fitness due to the symbiosis results in rapid population growth, dispersal and a greater potential for damage to soybeans and other legume crops [4]. The invasive also has a refuge, kudzu, which is now established in rural and urban regions across the entire southeastern United States [4]. This indicates that it will continue to be an urban nuisance pest as well as an agricultural threat. Unlike other stinkbug invasions [13,14], since the discovery of *M. cribraria* in 2009 entomologists have acted quickly to: identify and verify the taxonomy of the stinkbug and its primary endosymbiont [2,5]; study the natural history of the invasive [2,4]; and, use the insect's phenotype to track its temporal and spatial dispersal patterns [4]. We report the first genetic study of the Asian stinkbug. Mitochondrial DNA (mtDNA) and nuclear DNA (nuDNA) sequence data were collected for the purpose of developing an initial genetic baseline for the insect and the endosymbionts which have been shown to affect herbivory and survival [3,15]. The objectives of the work were to: determine the number of *M. cribraria* maternal lineages, track dispersal patterns and population structure of maternal lineages, and test the hypothesis that the obligate endosymbionts in the invasive insect host were the same as the obligate endosymbionts reported for *Megacopta* species in Japan [10,11,12,15].

## 2. Materials and Methods

### 2.1. Stink Bugs

Adult samples were collected in infested counties in Georgia and South Carolina and placed in 95%–100% EtOH. Each collection was labeled as to the date, location, and the collector's name.

### 2.2. DNA Extraction, Amplification and Sequencing

DNA was extracted from individual bugs preserved in 95%–100% EtOH with the DNeasy Blood & Tissue Kit (QIAGEN Inc., Valencia, CA). Extractions were performed in accordance with the protocols included with the kit.

A mitochondrial DNA (mtDNA) fragment (2336 bp) was amplified and sequenced in both directions from each of 83 samples collected from 36 counties in Georgia and two counties in South Carolina using two pairs of primers. This fragment consisted of the tyrosine tRNA, the cytochrome c oxidase subunit I gene (COI), the leucine tRNA, and the cytochrome c oxidase subunit II gene (COII). The 5′ end of the fragment was amplified and sequenced with primer MC-COI-Fwd (5′– ATGCCCAACTTCAGAATTGC–3′) as the forward primer and primer MC-COI-Rev (5′– ACTGCTCCTATGGATAATACG–3′) as the reverse primer. The 3′ end of the fragment was amplified and sequenced with primer MC-CO2-Fwd (5′–CTATTCACAATCGGAGGACTAA–3′) as the forward primer and primer MC-CO2-3700 (5′–GGTTTAAGAGACCAATGCT–3′) as the reverse primer.

In addition, two genes from the bacterial genome of the gammaproteobacteria were also partially amplified and sequenced with forward and reverse primers: 16S rRNA (1420 bp from 43 individuals), and *gro*EL (1554 bp from 35 individuals). The 16S rRNA gene primers were forward, 16SA1 (5′– AGAGTTTGATCMTGGCTCAG–3′) and reverse, 16SB1 (5′–TACGGYTACCTTGTTACGACTT– 3′) [3]. The *gro*EL chaperone gene was amplified and sequenced with primer Gro-F1 (5′–ATGGCAGCWAAAGACGTAAATTYGG–3′) as the forward primer and primer Gro-R1 (5′–TTACATCATKCCGCCCATGC–3′) as the reverse primer [11]. Lastly, the outer surface protein gene, *wsp*, of *Wolbachia* strains (552 bp from 35 individuals) were amplified and sequenced with primer MC-wsp-Fwd (5′–CAACGGTGAATTTTTACCTCT–3′) as the forward primer and MC-wsp- Rev (5′– GCTGTAAAGAACTTTGTATGCG–3′) as the reverse primer.

PCR was performed with a standard 25 μL reaction containing 5–20 ng of total genomic DNA. The reactions for each of the five amplifications had 1 pmol of each primer, 2.0 mM MgCl_2_, 1.0 mM dNTPs, and 0.06 U/μL *Taq* DNA polymerase. DNA was amplified in a Perkin-Elmer Gene Amp PCR system 9600 or 9700 (Applied Biosystems, Foster City, CA). The procedure included a pre-cycle denaturation at 94 °C for 2 min., a post-cycle extension at 72 °C for 7 min, and 35 cycles of a standard three-step PCR. The first and third steps were the same for each primer pair (94 °C for 1 min and 72 °C for 2 min). The second step varied for each primer pair: 53 °C for 1 min for the two mitochondrial primer pairs, 54 °C for 1 min for the 16SrRNA primer pair, 57 °C for 1 min for the *gro*EL primer pair, and 51 °C for 1 min for the *wsp* primer pair. PCR products were further purified using the QIAquick PCR Purification Kit (QIAGEN Inc., Valencia, CA) in accordance with the included protocol. All PCR samples were then sent to Eurofins MWG Operon (2211 Seminole Drive, Huntsville, Alabama 35805) for direct sequencing in both directions.

To further confirm maternal haplotype data, the entire mitochondrial genome (15,647 bp) was sequenced from five randomly chosen individuals collected from each of four counties where *M. cribraria* was first identified: one from Clark, one from Oconee, two from Hall and one from Jackson (Figure 1).

### 2.3. Sequence Analysis

All sequences were analyzed and initially aligned using Sequencher 4.0.1 software (Gene Codes Corp., Ann Arbor, MI, USA) according to established protocol [16]. Sequences were then aligned using CLUSTALW 1.83 [17]. A GenBank BLASTn search was also done on sequences to determine similarity to sequences in the sequence databases under the auspices of the International Nucleotide Sequence Database Collaboration (INSDC).

## 3. Results

### 3.1. Stink Bug Maternal Lineage

The 2336 bp mtDNA fragment which was sequenced from 83 individuals collected randomly from 36 counties in Georgia and two counties in South Carolina was the same for all individuals. The haplotype was designated GA1 (GenBank HQ444175). The mitochondrial genome sequences (15, 647 bp), which included the GA1 haplotype, from each of five individuals collected in four of the counties where the stink bug was initially identified (Figure 1) was the same for all five (GenBank No. JF288758).

### 3.2. Endosymbiont Genetic Identification

In order to develop a genetic baseline for this insect, the genetics of the endosymbiont had to be considered since the insect's survival depended on the bacteria [3]. To gain insight into the adaptive potential, biological history and fitness of the inherent symbiotic relationships two endosymbionts were studied: the obligate γ-proteobacterium, *Candidatus Ishikawaella capsulata*, necessary for *Megacopta* to thrive [11] and the *Wolbachia* α-proteobacterium, implicated in reproductive anomalies [18] as well as a mutualistic provider of essential nutrients [19].

#### 3.2.1. Candidatus Ishikawaella Capsulata

No variation among *C. I. capsulata* gene sequences from collections in Georgia and South Carolina was observed. The endosymbiont 16S rRNA gene sequence (GenBank No. JF732916) was 100% identical to all endosymbiont sequences from Georgia collections and to *Candidatus Ishikawaella capsulata* γ-proteobacteria from *Megacopta punctatissima* collected across south and central Japan, GenBank Nos. AB067723, AB244765, AB244766, AB240157 respectively [3,10]. The *C. I. capsulata gro*EL gene amplified from all GA1 samples (GenBank No. JF736508) was the same for all samples as well as 99% identical to the *C. I. capsulata* gene amplified from Japanese *M. cribraria* (GenBank No. AB109596) and *M. punctatissima* (GenBank No. AB109602) respectively.

#### 3.2.2. Wolbachia Species

Like the *C. I. capsulata* 16S rRNA and *gro*EL sequences, the *wsp* gene sequence was identical for all Georgia and South Carolina samples and 100% identical to *Wolbachia* strains amplified from *Megacopta punctatissima* in Japan (GenBank No. AB109596) [16]. Additionally, a pinned sample collected by G. E. Bohart in December 1952 from the Japanese Ryuku Islands in the East China Sea approximately within 120 km of Taiwan was sent to our lab by David Rider. The taxonomy of the sample in 1952 was identified as *Coptosoma cribraria*, but has sense been changed to *Megacopta cribraria* [2]. This museum specimen had the same *wsp* sequence as the sequences amplified from all individuals in Georgia, maternal haplotype GA1, and from the above *M. punctatissima* from Japan (GenBank No. AB109596).

## 4. Discussion

### 4.1. Stink Bug Maternal Lineage

The 2336 bp gene fragment from 83 individuals collected from 36 counties and the entire mtDNA genome (15, 647 bp) from five individuals collected from four of the original counties in which the bug was first discovered all had identical DNA sequence. These data strongly suggest that a single female lineage, GA1, has been introduced into North Georgia. The insect has been observed flying and landing on people as well as on and in their vehicles [4]. This phenomenon may have contributed to *M. cribraria*, GA1, rapidly dispersing throughout Georgia and into the contiguous state of South Carolina. If the insect continues to expand its range, it will likely disperse into other contiguous states (Figure 1). It has been able to overwinter in north Georgia where the temperature often fell below freezing between 2009 and 2010 [4]. Thus cold temperatures may not be a deterrent to dispersal. *Megacopta cribraria* was observed in June and July of 2010 feeding on kudzu vine, which was introduced into the United States from Japan in 1876 and has since spread across the southeastern United States [20]. It was also observed feeding on soybean plants at a density of ≥50 insects per plant [4].

*Megacopta cribraria* has shown that it is tolerant of some cold since it overwintered in north Georgia where there were days when temperatures fell below freezing between 2009 and 2010 [4]. The lack of genetic diversity observed in the mtDNA sequence is indicative of selection on this genome [21], which could be the result of multiple phenomena. The insect's ability to adapt across varied ecologies may be due to mutations in the nuclear genome resulting from the founder effect. These mutations created or exacerbated disequilibrium between the mtDNA and the nuclear genomes, which exerted strong selection pressure on a particular mtDNA sequence. A linkage disequilibrium between the mtDNA and nuclear genomes, or selection pressure from one or more of the endosymbiont genomes for a female lineage may also favor one mtDNA lineage over another. Each phenomenon could strengthen the bug's adaptive potential and facilitate expansion into novel geographies at least across the southeastern U. S.

### 4.2. Endosymbionts

Evidence from gene sequences amplified from individual *M. cribraria* of haplotype GA1 collected across counties in Georgia and South Carolina revealed two endosymbionts in the host stink bug: *Candidatus Ishikawaella capsulata*, a *γ* -proteobacterium in the order *Enterobacteriales* and *Wolbachia*, an α-proteobacterium in the order *Rickettsiales*.

*Candidatus Ishikawaella capsulata* are vertically inherited bacteria found in the midgut of *Megacopta* stink bugs in Japan [10]. These extracellular, primary symbionts [21] are maternally transmitted to offspring in capsules which are deposited on the underside of egg masses. When the nymphs hatch they quickly absorb the bacteria [10]. The *C. I. capsulata* 16S rRNA consensus sequence amplified from the GA1 samples aligned exactly with the 16S rRNA *C. I. capsulata* sequences amplified from *M. punctatissima* in Japan and recently deposited into GenBank. The *groEL* sequences deposited in GenBank (JR736508) showed 99% identity with *C. I. capsulata* amplified from both *M. cribraria* and *M. punctatissima* in Japan. This γ-proteobacterium in *M. cribraria* haplotype GA1, as in other hemiptera [22], keeps the insect alive and reproducing by synthesizing essential amino acids and other nutrients its food source does not provide [10]. Since the basic reproductive and nutritional needs of the stink bug are being met, female fecundity is likely to continue to be high exacerbating dispersal into new ecologies.

Although a single insect has been shown to be infected with multiple strains of *Wolbachia* [16], only one strain of *Wolbachia* was amplified from the plataspid insects in Georgia. This *Wolbachia* amplified from *M. punctatissima* collected in Japan and deposited in GenBank (AB109596) in 2003 had identical *wsp* sequence to those amplified from GA1 samples. It appears, therefore, that the *Wolbachia* bacterium found in the invasive stink bugs in Georgia is found in a different *Megacopta* species indigenous to Japan. This observation strongly suggest that more research needs to be done on the significance of horizontal transfer of the endosymbiont to other species within the genus, particularly since these accessory or facultative microorganisms have significant selective effects on reproduction, adaptation across environments, and dispersal [23].

## 5. Conclusions

The sequence data shows that only one female mitochondrial lineage, GA1 (HQ444175; JF288758), was introduced into Georgia and is rapidly dispersing throughout the state and into contiguous states. The data also show that the endosymbionts amplified from the *Megacopta* stink bugs in Georgia are also found in the *Megacopta* stink bugs in Japan. Two, vertically transmitted endosymbionts, the primary, extracellular *C. I. capsulata* (JF732916; JF736508) and the secondary, intracellular *Wolbachia* (AB109596), were amplified from all GA1 samples. *Megacopta cribraria* collected from kudzu to date appear to be approximately equal for males and females. This argues against a *Wolbachia*-associated cytoplasmic incompatibility [24] which could account for the sweep of a single mitochondrial genome [23]. It argues instead for a possible *Wolbachia*-associated nutritional mutualist [19]. The lack of maternal diversity may be indicative of indirect endosymbiont selection on the host. An insect-endosymbiont linkage disequilibrium [23] resulting in selection on the mtDNA could account for the observation of only one stink bug maternal haplotype and needs to be investigated further. Research into the question of mitochondrial-endosymbiont disequilibrium and its adaptive potential is needed to provide insights for effective and environmentally benign methods for managing the insect.
